# Dietary Supplementation with *Lactobacillus casei* Alleviates Lipopolysaccharide-Induced Liver Injury in a Porcine Model

**DOI:** 10.3390/ijms18122535

**Published:** 2017-11-26

**Authors:** Di Zhao, Tao Wu, Dan Yi, Lei Wang, Peng Li, Junmei Zhang, Yongqing Hou, Guoyao Wu

**Affiliations:** 1 Hubei Collaborative Innovation Center for Animal Nutrition and Feed Safety, Hubei Key Laboratory of Animal Nutrition and Feed Science, Wuhan Polytechnic University, Wuhan 430023, China; zhaodi@whpu.edu.cn (D.Z.); wutao@whpu.edu.cn (T.W.); yidan810204@whpu.edu.cn (D.Y.); wanglei@whpu.edu.cn (L.W.); Lipeng@whpu.edu.cn (P.L.); zhangjm@whpu.edu.cn (J.Z.); g-wu@exchange.tamu.edu (G.W.); 2 Department of Animal Science, Texas A & M University, College Station, TX 77843, USA

**Keywords:** *Lactobacillus casei*, piglets, lipopolysaccharide, liver

## Abstract

This study aims to determine whether *Lactobacillus casei* (*L. casei*) could relieve liver injury in piglets challenged with lipopolysaccharide (LPS). Piglets were randomly allocated into one of the three groups: control, LPS, and *L. casei*. The control and LPS groups were fed a corn- and soybean meal-based diet, whereas the *L. casei* group was fed the basal diet supplemented with 6 × 10^6^ cfu/g *L. casei*. On Day 31 of the trial, piglets in the LPS and *L. casei* groups received intraperitoneal administration of LPS (100 µg/kg body weight), while the control group received the same volume of saline. Blood and liver samples were collected for analysis. Results showed that *L. casei* supplementation decreased the feed/gain ratio (*p* = 0.027) and diarrhea incidence (*p* < 0.001), and attenuated LPS-induced liver histomorphological abnormalities. Compared with the control group, LPS challenge dramatically increased glutamyl transpeptidase activity (*p* = 0.001) in plasma as well as the concentrations of Interleukin 6 (IL-6) (*p* = 0.048), Tumor necrosis factor-alpha (TNF-α) (*p* = 0.041), and Malondialdehyde (MDA) (*p* = 0.001) in the liver, while decreasing the hepatic SOD activity. LPS also increased (*p* < 0.05) the mRNA levels for IL-6, IL-8, TNF-α, Toll-like receptors 4 (TLR4), Nuclear factor κB (NF-κB) and Heat shock protein 70 (HSP70) in the liver. The adverse effects of LPS challenge were ameliorated by *L. casei* supplementation. In conclusion, dietary *L. casei* alleviates LPS-induced liver injury via reducing pro-inflammatory cytokines and increasing anti-oxidative capacity.

## 1. Introduction

As a major site for nutrient metabolism and detoxification in the body, the liver plays a critical role in preventing exogenous toxic substances from entering the systemic blood stream [1]. Factors such as bacterial and viral infection or inflammation lead to the activation of macrophages (Kupffer cells), which results in increased productions of pro-inflammatory cytokines (e.g., tumor necrosis factor (TNF)-α, interleukine (IL)-1β and IL-6) and reactive oxygen species (ROS) [2], and consequently parenchymal liver damage and dysfunction. Lipopolysaccharide (LPS), a component of the outer membrane of Gram-negative bacteria, is known to stimulate Kupffer cells (macrophages) and result in inflammatory liver injury [3].

*Lactobacillus casei* (*L. casei*) is regarded as a probiotic and is widely used in the food industry [4]. This beneficial bacterium has attracted the focus of research due to its potential immunoregulatory effect. It has been demonstrated that specific *L. casei* could modulate host immunity, which is positively correlated with the enhanced resistance to various viral and bacterial infections [4]. The immunomodulatory effects are dependent on various factors, such as the intrinsic adjuvant properties, dose, viability, route and timing of administration of the specific *L. casei*, as well as the physiological state and genetic background of the host [5,6,7]. However, literature concerning the effects of *L. casei* on liver inflammation and injury is scarce. Therefore, the present study was conducted to determine whether *L. casei* could attenuate liver injury by using a piglet model with LPS challenge [6].

## 2. Results

### 2.1. Growth Performance

During Days 1–30 of the trial, dietary supplementation with *L. casei* decreased the ratio of feed to gain (F/G) and diarrhea incidence of piglets, in comparison with the control group (Table 1).

### 2.2. Liver Histology

**Figure 1:**
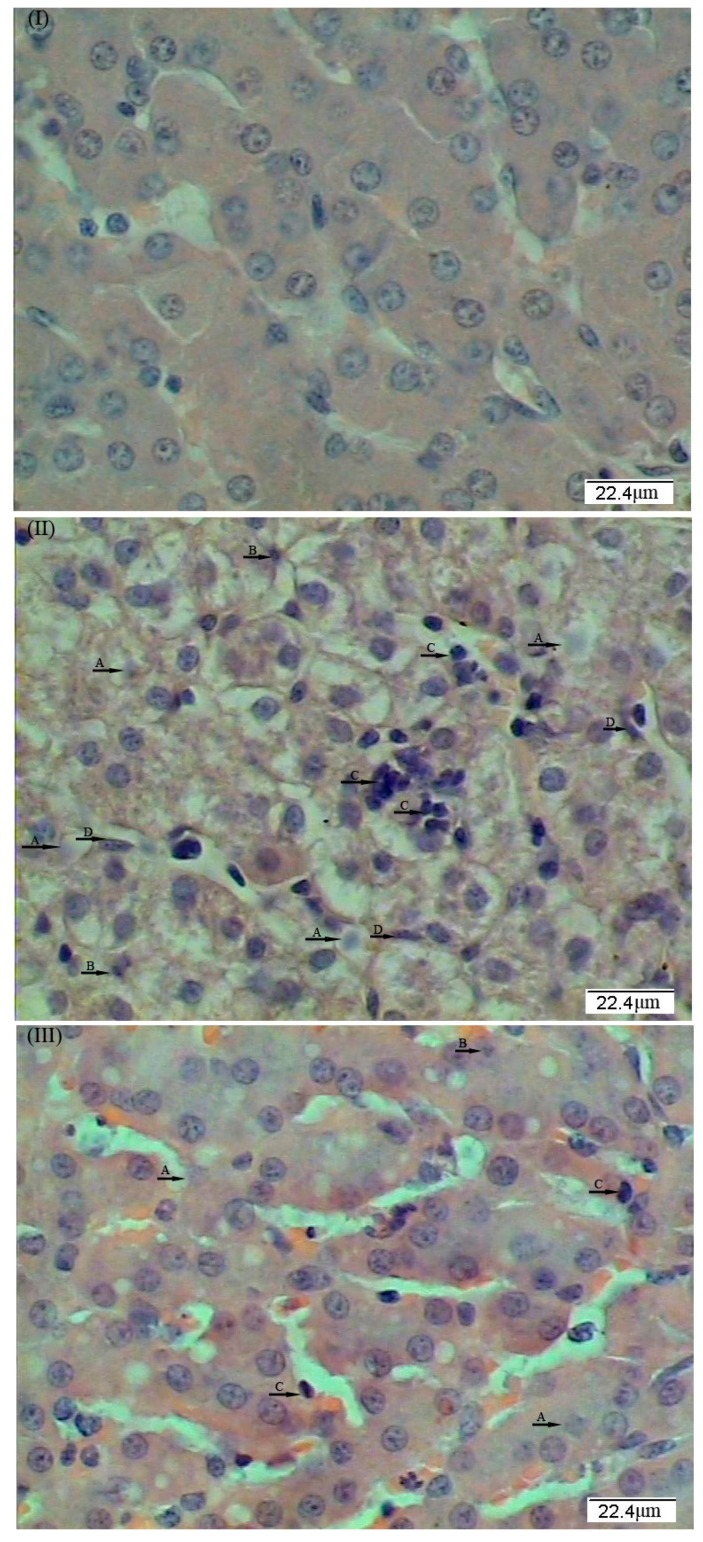
Effects of dietary supplementation with *L. casei* on liver morphology. Representative photomicrographs of liver sections stained with hematoxylin and eosin are shown (×400): (**I**) piglets fed the basal diet and received intraperitoneal administration of sterile saline; (**II**) piglets fed the basal diet and received intraperitoneal administration of *Escherichia coli* lipopolysaccharide (LPS); and (**III**) piglets fed the basal diet supplemented with 6 × 10^6^ cfu/g *L. casei* and received intraperitoneal administration of LPS, as described in the Material and Methods Section. Histological analysis revealed abnormalities in hepatic structure, including: caryolysis (A); karyopycnosis (B); infiltration of inflammatory cells (C); injury to hepatic sinusoids (D); and the disordered arrangement of hepatic cell cords.

The morphological structure of the liver is shown in Figure 1. The livers of piglets in the control group (Figure 1I) appeared to be normal. The intraperitoneal administration of LPS induced histopathological changes in the liver, including: (A) the infiltration of inflammatory leukocytes into the hepatic lobes; (B) the caryolysis, karyopycnosis, and vacuolation of hepatocytes; (C) the disordered arrangement of hepatic cell cords; and (D) hemorrhage (Figure 1II). The LPS-induced liver injury was attenuated by dietary supplementation with *L. casei*, as indicated by the normal hepatic structure, the mild vacuolation of hepatic cells, and limited infiltration of inflammatory leukocytes (Figure 1III).

### 2.3. Alanine Aminotransferase (ALT), Aspartate Aminotransferase (AST), Glutamyl Transpeptidase (GGT) Activity in Plasma

The activities of ALT, AST, GGT in plasma are shown in Table 2. LPS challenge increased (*p* < 0.05) AST and GGT activities in plasma, compared with the control group. Dietary supplementation with *L. casei* decreased the blood GGT (*p* < 0.05) activity in LPS-challenged piglets, despite no effects on ALT and AST activities.

### 2.4. IL-6 and TNF-α Concentrations in the Liver

As shown in Table 3, LPS challenge increased (*p* < 0.05) the concentrations of IL-6 and TNF-α in the liver of piglets in comparison with the control group. However, dietary supplementation with *L. casei* decreased (*p* < 0.05) the concentrations of those two pro-inflammatory cytokines in the liver of LPS-challenged piglets (Table 3).

### 2.5. Liver Redox Status

The concentrations of MDA and H_2_O_2_, as well as the enzymatic activities of Superoxide dismutase (SOD), Catalase (CAT), and Glutathione peroxidase (GSH-Px), in the liver are summarized in Table 4. LPS challenge reduced (*p* < 0.05) SOD activity but increased MDA levels in the liver, as compared with the control group. However, dietary supplementation with *L. casei* attenuated the reduction in hepatic SOD activity (*p* < 0.05) and the increase in hepatic MDA concentrations in LPS-challenged piglets. The activities of CAT and GSH-Px, and H_2_O_2_ levels in the liver were not affected by *L. casei* supplementation (*p* > 0.05).

### 2.6. IL-6, IL-8, TNF-α, TLR4, NF-κB and HSP70 mRNA Expression in the Liver

Data on mRNA levels for hepatic *IL-6*, *IL-8*, *TNF-α*, *TLR4*, *NF-κB* and *HSP70* are summarized in Table 5. Compared with the control group, LPS-challenged piglets had higher (*p* < 0.05) mRNA levels for *IL-6*, *IL-8*, *TNF-α*, *TLR4*, *NF-κB*, and *HSP70* in the liver. However, the increases in hepatic *IL-6*, *IL-8*, *TNF-α*, *NF-κB*, and *HSP70* were attenuated (*p* < 0.05) in piglets receiving the *L. casei*-supplemented diet. The mRNA expression of hepatic *TLR4* was not altered by *L. casei* supplementation (*p* > 0.05). 

## 3. Discussion

In this study, to investigate whether dietary supplementation of *L. casei* could alleviate liver injury, we utilized a well-established porcine model with LPS-induced hepatic damage. In this animal model, liver injury was induced by intraperitoneal administration of *E. coli* LPS. LPS, which commonly exists in the outer membrane of all Gram-negative bacteria, can bind to and activate the Kupffer cells (specialized macrophages located in the liver), resulting in the enhanced release of pro-inflammatory cytokines [4,5]. This LPS-induced liver injury model has been commonly used to elucidate the mechanism of inflammatory liver injury and the protective effects of nutritional ingredients, such as probiotics and amino acids [6,8,9,10].

The dose of *L. casei* used for the present study was based on the results of our previous work regarding the effects of its dietary supplementation on LPS-induced liver injury on improving the growth performance of piglets [11]. Interestingly, piglets fed the *L. casei* diet exhibited a lower feed/gain ratio and a lower rate of diarrhea in comparison with those fed the basal diet. Diarrhea commonly occurs in early-weaned piglets because of their intestinal dysfunction in response to various challenges, such as social, environmental and dietary stresses [6]. In *L. casei*-supplemented piglets, reduced diarrhea incidence indicates an improvement in intestinal health. As a probiotic, *L. casei* has been reported to enhance intestinal-mucosal barrier function and immunity [12] and modulate the intestinal ecology [11,12,13].

Plasma ALT, AST, and GGT activities are sensitive markers for hepatic damage [2]. Results of the present study showed that dietary supplementation with *L. casei* attenuated LPS-induced increases in plasma GGT activities, indicating a positive beneficial effect of *L. casei* in ameliorating liver injury. These results are consistent with the histopathological changes in liver morphology, which demonstrated that dietary *L. casei* supplementation mitigated the LPS-induced damage to the hepatic architecture. However, results on plasma ALT and AST activities indicated an incomplete recovery of the liver from LPS challenge, which may be due to the dosage of *L. casei* (1 × 10^8^ cfu/day per kg body weight) used in the present study. Of note, a higher dosage of *L. casei* (6.8 × 10^10^ cfu/day per kg body weight) could restore the plasma ALT activity to the normal level and protect the liver from fructose-induced steatosis in mice [14]. Further studies are warranted to determine dose-dependent effects of *L. casei* on hepatic structure and function.

LPS stimulated the release of inflammatory cytokines, which consequently induced the production of ROS and related peroxides, and ultimately resulted in the aggravation of the liver injury [10]. However, the liver possesses defensive mechanisms against ROS through the actions of radical scavengers, such as SOD, CAT, and GSH-Px [15]. In the present study, the activity of SOD in the liver was much lower in the LPS group than in the control group. Of note, dietary supplementation with *L. casei* effectively mitigated the oxidative damage caused by LPS. Lactobacillus bacteria act through various mechanisms to defend animals against ROS toxicity, such as synthesizing SODs, producing hydroperoxidases, and accumulating high intracellular levels of metal ions. SOD defends against oxidative stress by scavenging O_2_^−^ into O_2_ and H_2_O_2_, whereas catalase decomposes H_2_O_2_ into H_2_O and O_2_. It is likely that dietary supplementation with *L. casei* improved the liver health in piglets through increasing the activity of SOD to protect against oxidative damage. Similarly, Wang et al. [13] found that pretreatment with *L. casei* significantly increased SOD activity in the homogenates of the liver challenged with LPS. Taken together, these findings support the notion that *L. casei* is an effective agent for enhancing the anti-oxidative capacity in animals.

LPS also induces the production of pro-inflammatory cytokines by hepatic Kupffer cells via activating pattern recognition through toll-like receptors (TLRs) [10]. Among the identified TLRs, TLR4 is a well defined receptor for LPS recognition. Upon activation, the TLR4 signaling drives Kupffer cells to produce a variety of inflammation-related cytokines [16]. The nuclear regulatory factor κB (NF-κB) is a central regulator of cellular stress in all cell types in the liver. NF-κB activation is triggered via canonical pathways in response to a wide variety of stimuli, including pro-inflammatory cytokines, as well as the bacterial and viral antigens that act on TLRs [17]. Moreover, NF-κB is a family of dimeric transcription factors that regulate inflammation, innate and adaptive immunity, and wound healing responses, as well as cell fate and function [18]. NF-κB plays these physiological roles by binding to κB sequences found in the regulatory regions of more than 200 target genes. The elevated abundance of NF-κB in the liver plays a mediatory role in the stimulatory effect of LPS on the production of inflammatory cytokines (IL-6 and TNF-α) in piglets. To support this notion, we observed an increase in the concentrations of hepatic IL-6 and TNF-α in LPS-challenged piglets (Table 3). In addition, NF-κB regulates the expression of many downstream genes that control cell proliferation, survival, stress responses, and immunity. Under LPS challenge, *TLR4* and *NF-κB* mRNA levels were markedly enhanced, suggesting that TLR4 and NF-κB signaling pathways may be activated by LPS. However, the diet supplemented with *L. casei* reduced the hepatic concentrations of IL-6 and TNF-α, as well as the hepatic mRNA levels for pro-inflammatory cytokines (e.g., *IL-6* and *IL-8*) in LPS-challenged piglets (Table 5). Collectively, these results indicate that dietary supplementation with *L. casei* attenuated the hepatic inflammation possibly via activating the TLR4/NF-κB signaling pathway.

In response to stresses, the mRNA abundance of hepatic heat shock protein HSP70 is usually enhanced to promote the refolding of partially-denatured proteins and prevent their aggregation, thereby protecting cells from injury [8]. This is an adaptive mechanism for allowing organisms to survive heat shock stress. Therefore, a high level of *HSP70* is a sensitive indicator of oxidative stress in tissues [19]. In the present study, the expression of the hepatic *HSP70* gene was dramatically increased in the liver of LPS-challenged piglets, but was decreased when the diet was supplemented with *L. casei.* These results further support the notion that *L. casei* plays an important role in ameliorating liver oxidative stress.

In summary, dietary supplementation with 6 × 10^6^ cfu/g *L. casei* exerts beneficial effects in alleviating liver injury in lipopolysaccharide-challenged piglets. The hepato-protective effects of *L. casei* is closely associated with its role in increasing anti-oxidative capacity and reducing pro-inflammatory cytokines in the liver of piglets. These novel findings have important implications for improving the nutritional status of infected animals. As the piglet is a well-established animal model for studying human nutrition and disease [6], findings from the porcine model may be used for the treatment of human liver disease.

## 4. Material and Methods

### 4.1. Experimental Design

The animal use protocol for this research (2012-0820, 11 September 2012) was approved by the Institutional Animal Care and Use Committee at Wuhan Polytechnic University. Eighteen healthy crossbred female piglets (Duroc × Landrace × Yorkshire), which were reared by sows, were weaned at 21 ± 2 days of age. After a 4-day period of adaptation, piglets (25 ± 2 days of age, average body weight of 6.40 ± 0.53 kg) were housed individually in stainless steel metabolic cages (1.20 × 1.10 m^2^) and maintained in an environmentally controlled room (25 °C) by air conditioning. Piglets had free access to food and water. The corn and soybean meal-based diet (Table 6) was formulated to meet National Research Council’s (NRC, 2012) recommended requirements for all nutrients [20]. The dietary content of crude protein (CP), calcium (Ca), and total phosphorus (P) was analyzed according to the Weende method of the feed proximate analysis as described by Henneberg and Stohmann [21]. The dietary content of total lysine, methionine, cystine, threonine, and tryptophan was analyzed by automatic amino acids analyzer (S433D, Sykam GmbH, Eresing, Germany) [22].

All piglets had free access to the basal diet during a 4-day adaptation period to help them adapt to solid food. On Day 1 of the trial (25 days of age), piglets were assigned randomly into three groups (6 piglets/group): (1) control group (piglets fed the basal diet and received intraperitoneal treatment with sterile saline); (2) LPS group (piglets fed the basal diet and received intraperitoneal treatment with *Escherichia coli* LPS); and (3) *L. casei* group (LPS + 6 × 10^6^ cfu/g *L. casei*, piglets fed the basal diet supplemented with 6 × 10^6^ cfu/g *L. casei* and received intraperitoneal administration of *Escherichia coli* LPS). LPS was dissolved in sterile saline. *L. casei* (powder) was well mixed with the basal diet in a one-batch mixing. The dosage of 6 × 10^6^ cfu/g *L. casei* was chosen because it was found in our previous study to be effective in improving the growth performance and intestinal function of piglets [11]. *L. casei* was grown overnight at 37 °C in MRS broth (HB0384-1; Oxoid, Haibo, China) and centrifuged at 3000 rpm for 10 min at room temperature, then a *L. casei* powder with a concentration of 2 × 10^11^ cfu/g was obtained by vacuum drying. The *L. casei* powder was mixed with feed to a final concentration of 6 × 10^6^ cfu/g. On Day 31 of the trial, overnight fasted piglets in the LPS and *L. casei* groups received an intraperitoneal injection of LPS (*Escherichia coli* serotype O55: B5; Sigma Chemical Inc., St. Louis, MO, USA) at the dose of 100 µg/kg BW, whereas piglets in the control group received an intraperitoneal injection of the same volume of sterile saline [23]. At 6 h post-injection of LPS or saline, all piglets were killed by an intravenous injection of pentobarbital sodium (50 mg/kg BW) and liver samples were collected [8]. During Days 1–30 of the trial, the body weight, feed intake, and diarrhea incidences of piglets were recorded to statistically analyze their growth performance.

### 4.2. Blood Sample Collection

On Day 31 of the trial, blood samples were collected from the anterior vena cava into heparinized vacuum tubes at 3 h post LPS or saline injection (Becton Dickinson Vacutainer System, Franklin Lake, NJ, USA). Blood samples (7 mL) were centrifuged at 1000× *g* for 10 min at 4 °C to separate plasma and then stored at −80 °C for further analysis.

### 4.3. Liver Samples Collection and Histology Analysis

The abdomen of piglets was surgically opened immediately from the sternum to the pubis, and the liver without the cholecyst was collected. A liver sample (~5 g) was collected from the left lobe of the liver and rinsed thoroughly with ice-cold phosphate buffered saline (PBS, pH = 7.4) to remove blood contamination. Liver samples were then rapidly frozen in liquid nitrogen and stored at −80 °C for further analysis. All samples were collected within 15 min after euthanasia.

For histomorphological analysis of liver samples, the 0.5 cm^3^ segments were cut off from the liver, and flushed with ice-cold PBS. Liver segments were then fixed in fresh 4% paraformaldehyde/phosphate-buffered saline and embedded in paraffin, sectioned at 5 µm and stained with hematoxylin and eosin [8]. Histomorphological examination was performed with a light microscope (American Optical Co., Scientific Instrument Div., Buffalo, NY, USA).

### 4.4. ALT, AST, GGT Activity in Plasma

Alanine aminotransferase (ALT), aspartate aminotransferase (AST), and glutamyl transpeptidase (GGT) in plasma were assayed by a Hitachi automatic biochemistry analyzer 7100 with WAKO chemical reagents (Wako Pure Chemical Industries, Ltd., Osaka, Japan). 

### 4.5. IL-6 and TNF-α Levels in the Liver

Concentrations of pro-inflammatory cytokines in the liver were determined as previously described [17]. Briefly, frozen liver samples were homogenized in ice-cold PBS-EDTA buffer (0.05 mol/L Na_3_PO_4_, 2.0 mol/L NaCl, 2 mmol/L EDTA, pH 7.4), and the homogenates were centrifuged to obtain the supernatant fluid. Tumor necrosis factor-α (TNF-α) and Interleukin-6 (IL-6) in the liver supernatant fluid were analyzed using commercially available ^125^I kits (Beijing North Institute of Biological Technology, Beijing, China). The detection limit for TNF-α was 0.03 ng/mL, and the intra- and interassay coefficients of variation were 5% and 8%, respectively. The detection limits for IL-6 analyses were 5.0 pg/mL, and the coefficients of variation for intra- and inter-assays of IL-6 were less than 7% and 15%, respectively.

### 4.6. MDA, H_2_O_2_, SOD, CAT and GSH-Px Activity in Liver

The liver tissue (~200 mg) was homogenized in a nine-fold volume of freezing saline, and then centrifuged at 2500 rpm for 10 min at 4 °C to obtain the supernatant fluid used for assays. Malondialdehyde (MDA), H_2_O_2_, superoxide dismutase (SOD), catalase (CAT), and glutathione peroxidase (GSH-Px) in the liver were determined using commercially available kits (Nanjing Jiancheng Bioengineering Institute, Nanjing, China).

### 4.7. IL-6, IL-8, TNF-α, TLR4, NF-κB, and HSP70 mRNA Expression

mRNA levels for Interleukin-6 (IL-6), interleukin-8 (IL-8), tumor necrosis factor-alpha (TNF-α), toll-like receptor 4 (TLR4), nuclear factor kB (NF-κB), and heat shock protein 70 (HSP70) in the liver were quantified using quantitative RT-PCR, as described by Hou et al. [17]. A frozen liver sample of approximately 100 mg was powdered under liquid nitrogen using a mortar and pestle, then homogenized in a buffer. Total RNA was isolated using the TRIzol Reagent protocol (Cat. 15596026, Invitrogen, Carlsbad, CA, USA), and was quantified using the NanoDrop^®^ ND-2000 UV–Vis spectrophotometer (Thermo Scientific, Wilmington, DE, USA) at 260 and 280 nm. The purity of RNA was assessed by determining the OD260/OD280 ratio. All of the samples had an OD260/OD280 ratio above 1.8, corresponding to 90–100% pure nucleic acids. Meanwhile, the integrity of RNA in each sample was assessed using 1% denatured agarose gel electrophoresis. RNA was used for quantitative RT-PCR analysis when the sample had a 28S/18S rRNA ratio ≥ 1.8 [24]. Total RNA was reverse-transcribed using the Prime-Script^®^ RT reagent kit with gDNA Eraser (Cat. RR047A, Takara, Dalian, China) according to the manufacturer’s instructions. cDNA was synthesized and stored at −20 °C for further analysis. The RT-PCR analysis of gene expression was performed using primers for *IL-6*, *IL-8*, *TNF-α*, *TLR4*, *NF-κB*, *HSP70*, *ribosomal protein L4* (*RPL4*) and *glyceraldehyde-3-phosphate dehydrogenase* (*GAPDH*) (Table 7), and the SYBR^®^ Premix Ex Taq™ (Cat. 420A, Takara, Dalian, China) on an Applied Biosystems 7500 Real-Time PCR System (Foster City, CA, USA). The total volume of the PCR reaction system was 20 µL. In brief, the reaction mixture contained 10.0 µL SYBR Premix ExTaq, 0.4 µL ROX reference dye II (50×), 2.0 µL cDNA, 6.8 µL RNase free water, 0.4 µL forward primer (10 µmol/L), and 0.4 µL reverse primer (10 µmol/L). All PCRs were done in triplicate on a 96-well RT-PCR plate under the following conditions (two-step amplification): 95 °C for 30 s, followed by 40 cycles of 95 °C for 5 s, and 60 °C for 34 s. A subsequent melting curve (95 °C for 15 s, 60 °C for 1 min and 95 °C for 15 s) with continuous fluorescence measurement and final cooling to 25 °C was processed. Amplification products were verified by melting curves and agarose gel electrophoresis. Each biological sample was run in triplicate. To ensure the sensitivity and accuracy of the results obtained by qPCR, samples were normalized internally using simultaneously *RPL4* and *GAPDH* as references in each sample to avoid any artifact of variation in the target gene. Results were analyzed by 2^−ΔΔ*C*t^ method [25].

### 4.8. Statistical Analysis

Data expressed as means ± SD were analyzed by one-way analysis of variance. The normality and constant variance for data were tested by the Levene’s test [26]. Differences among treatment means were determined by the Duncan’s post hoc test. All statistical analyses were performed by the SPSS 13.0 software (Chicago, IL, USA). Possibility values <0.05 were taken to indicate statistical significance [27].

## 5. Conclusions

Diet supplementation with 6 × 10^6^ cfu/g *L. casei* exerts beneficial effects in alleviating liver injury in lipopolysaccharide-challenged piglets. The hepato-protective effects of *L. casei* is closely associated with its role in increasing anti-oxidative capacity and reducing pro-inflammatory cytokines in the liver of piglets.

## Figures and Tables

**Table 1:** Effect of dietary supplementation with *L. casei* on the growth performance of piglets during Days 1–30 of the trial.

Items	Control Group	*L. casei* Group	*p*-Value
BW (kg)	6.33 ± 1.07	6.48 ± 1.28	0.840
ADG (g/day)	410 ± 51	412 ± 32	0.945
F/G ratio	1.82 ± 0.03 ^a^	1.75 ± 0.06 ^b^	0.027
Diarrhea incidence (%)	14.6 ± 2.63 ^a^	4.17 ± 0.83 ^b^	<0.001

Data are means ± SD, *n* = 12 for the control group and *n* = 12 for the *L. casei* group. BW = body weight; ADG = average daily gain; F/G = feed/gain ratio. Control group = piglets fed the basal diet; *L. casei* group = piglets fed the basal diet supplemented with 6 × 10^6^ cfu/g *L. casei*; ^a,b^ Values within a row with different superscript letters differ (*p* < 0.05).

**Table 2:** Effects of dietary supplementation with *L. casei* on activities of ALT, AST, GGT in the piglet plasma.

Items	Control Group	LPS Group	*L. casei* Group	*p*-Value
ALT (U/L)	43.4 ± 7.70	45.1 ± 7.53	48.8 ± 8.51	0.354
AST (U/L)	34.6 ± 6.78 ^b^	56.9 ± 12.8 ^a^	50.9 ± 7.06 ^a^	0.021
GGT (U/L)	27.2 ± 6.12 ^b^	46.5 ± 4.15 ^a^	27.8 ± 4.60 ^b^	0.001

Data are means ± SD, *n* = 6. ALT = alanine aminotransferase; AST = aspartate aminotransferase; GGT = glutamyl transpeptidase. Control group = piglets fed the basal diet and received administration of saline; LPS group = piglets fed the basal diet and challenged with LPS; *L. casei* group = piglets fed the basal diet supplemented with 6 × 10^6^ cfu/g *L. casei* and challenged with LPS; ^a,b^ Values within a row with different superscript letters differ significantly (*p* < 0.05).

**Table 3:** Effects of dietary supplementation with *L. casei* on the concentrations of IL-6 and TNF-α in the piglet liver.

Items	Control Group	LPS Group	*L. casei* Group	*p*-Value
IL-6 (pg/mg)	5.86 ± 0.33 ^b^	7.20 ± 0.31 ^a^	5.85 ± 0.79 ^b^	0.048
TNF-α (ng/mg)	0.15 ± 0.03 ^b^	0.20 ± 0.01 ^a^	0.15 ± 0.04 ^b^	0.041

Data are means ± SD, *n* = 6. IL-6 = Interleukin 6; TNF-α = tumor necrosis factor-alpha. Control group = piglets fed the basal diet and received administration of saline; LPS group = piglets fed the basal diet and challenged with LPS; *L. casei* group = piglets fed the basal diet supplemented with 6 × 10^6^ cfu/g *L. casei* and challenged with LPS; ^a,b^ Values within a row with different superscript letters differ significantly (*p* < 0.05).

**Table 4:** Effects of dietary supplementation with *L. casei* on the concentrations of MDA and H_2_O_2_, as well as the enzymatic activities of SOD, CAT, and GSH-Px, in the piglet liver.

Items	Control Group	LPS Group	*L. casei* Group	*p*-Value
SOD (U/mg protein)	86.7 ± 9.0 ^a^	69.8 ± 4.7 ^b^	91.4 ± 1.2 ^a^	0.002
CAT (U/g protein)	30.0 ± 4.5	28.3 ± 7.1	26.0 ± 6.1	0.559
GSH-Px (U/g protein)	117 ± 25.5	95.3 ± 25.3	108 ± 25.5	0.583
MDA (µg/g protein)	7.1 ± 1.5 ^b^	11.6 ± 1.7 ^a^	6.9 ± 0.6 ^b^	0.001
H_2_O_2_ (µg/g protein)	50.1 ± 11.1	56.7 ± 3.4	54.7 ± 13.4	0.818

Data are means ± SD, *n* = 6. MDA = malondialdehyde; H_2_O_2_ = hydrogen peroxide; SOD = superoxide dismutase; CAT = catalase; GSH-Px = glutathione peroxidase. Control group = piglets fed the basal diet and received administration of saline; LPS group = piglets fed the basal diet and challenged with LPS; *L. casei* group = piglets fed the basal diet supplemented with 6 × 10^6^ cfu/g *L. casei* and challenged with LPS; ^a,b^ Values within a row with different superscript letters differ significantly (*p* < 0.05).

**Table 5:** Effects of dietary supplementation with *L. casei* on mRNA levels for inflammatory genes in the liver of piglets.

Genes	Control Group	LPS Group	*L. casei* Group	*p*-Value
*IL-6*	1 ± 0.23 ^c^	9.27 ± 0.44 ^a^	5.17 ± 0.15 ^b^	0.004
*IL-8*	1 ± 0.32 ^c^	55.5 ± 10.1 ^a^	38.0 ± 3.36 ^b^	<0.001
*TNF-α*	1 ± 0.16 ^c^	6.43 ± 1.25 ^a^	3.02 ± 0.49 ^b^	0.001
*TLR4*	1 ± 0.25 ^b^	1.77 ± 0.08 ^a^	1.74 ± 0.01 ^a^	0.033
*NF-κB*	1 ± 0.04 ^b^	2.10 ± 0.32 ^a^	1.36 ± 0.23 ^b^	0.006
*HSP70*	1 ± 0.18 ^c^	20.4 ± 5.81 ^a^	10.4 ± 2.31 ^b^	0.017

All mRNA levels in the control group were regarded as 1. Data are means ± SD, *n* = 6. IL-6 = interleukin 6, IL-8 = interleukin 8, TNF-α = tumor necrosis factor-alpha, TLR4 = toll-like receptors 4, NF-κB = nuclear factor κB, HSP70 = heat shock protein 70. Control group = piglets fed the basal diet and received administration of saline; LPS group = piglets fed the basal diet and challenged with LPS; *L. casei* group = piglets fed the basal diet supplemented with 6 × 10^6^ cfu/g *L. casei* and challenged with LPS; ^a,b,c^ Values within a row with different superscript letters differ significantly (*p* < 0.05).

**Table 6:** Composition and nutrient contents of the basal diet (as-fed basis).

Ingredients	Content (%)
Corn (DE 14.27 MJ/kg, CP 8.7%)	61.88
Soybean meal (DE 13.5 MJ/kg, CP 40%)	21.98
Wheat Middling (DE 13.4 MJ/kg, CP 13%)	4.00
Fish meal (CP 66%)	3.00
Dried whey (CP 12%)	3.00
Soy protein concentrate (CP 65%)	1.50
CaHPO_4_	1.25
Premix ^†^	1.00
Limestone (CaCO_3_ > 35%)	0.69
Soy oil	0.50
Acidifier (Citric acid > 99%)	0.30
NaCl	0.30
Mould inhibitor (Calcium propionate > 30%)	0.10
Choline chloride	0.20
l-Lysine·HCl (78.8% lysine)	0.25
dl-Methionine (99% methionine)	0.05
Nutrients composition	
Digestible energy ^‡^ (MJ/kg)	14.22
Crude protein (%) ^§^	20.90
Total lysine (%) ^§^	1.15
Total methionine (%) ^§^	0.30
Total threonine (%) ^§^	0.74
Total tryptophan (%) ^§^	0.21
Total calcium (%) ^§^	0.70
Total phosphorus (%) ^§^	0.60
Available phosphorus (%) ^‡^	0.32

^†^ Premix provided the following amounts of vitamins and trace minerals per kilogram of the complete diet: ferrum, 100 mg (FeSO_4_·H_2_O); copper, 150 mg (CuSO_4_·5H_2_O); manganese, 40 mg (MnSO_4_·5H_2_O); zinc, 100 mg (ZnSO_4_·7H_2_O); iodine, 0.5 mg (KI); selenium, 0.3 mg (Na_2_SeO_3_·5H_2_O); vitamin A acetate, 3.66 mg; cholecalciferol, 0.10 mg; DL-α-tocopheryl acetate, 36.4 mg; menadione, 4 mg; thiamin, 6 mg; riboflavin, 12 mg; pyridoxine, 6 mg; cyanocobalamin, 0.05 mg; biotin, 0.2 mg; folic acid, 2 mg; niacin, 50 mg; d-calcium pantothenate, 25 mg; ^‡^ Calculated value; ^§^ Analyzed value.

**Table 7:** Sequences of the primers used for quantitative RT-PCR analysis.

Gene	Forward (5′–3′)	Reverse (5′–3′)
*IL-6*	TACTGGCAGAAAACAACCTG	GTACTAATCTGCACAGCCTC
*IL-8*	TTCGATGCCAGTGCATAAATA	CTGTACAACCTTCTGCACCCA
*TNF-α*	TCCAATGGCAGAGTGGGTATG	AGCTGGTTGTCTTTCAGCTTCAC
*TLR4*	GCCTTTCTCTCCTGCCTGAG	AGCTCCATGCATTGGTAACTAATG
*NF-κB*	CTCGCACAAGGAGACATGAA	ACTCAGCCGGAAGGCATTAT
*HSP70*	GACGGAAGCACAGGAAGGA	GAAGACAGGGTGCGTTTGG
*RPL4*	GAGAAACCGTCGCCGAAT	GCCCACCAGGAGCAAGTT
*GAPDH*	CGTCCCTGAGACACGATGGT	CCCGATGCGGCCAAAT

## Abbreviations

*L. casei*	*Lactobacillus casei*
LPS	Lipopolysaccharide
ALT	Alanine aminotransferase
AST	Aspartate aminotransferase
GGT	Glutamyl transpeptidase
MDA	Malondialdehyde
SOD	Superoxide dismutase
CAT	Catalase
GSH-Px	Glutathione peroxidase
IL-6	Interleukin 6
IL-8	Interleukin 8
TNF-α	Tumor necrosis factor-alpha
TLR4	Toll-like receptors 4
NF-κB	Nuclear factor κB
HSP70	Heat shock protein 70
